# Updated List of Transport Proteins in *Plasmodium falciparum*

**DOI:** 10.3389/fcimb.2022.926541

**Published:** 2022-06-24

**Authors:** Juliane Wunderlich

**Affiliations:** ^1^ Max Planck Institute for Infection Biology, Berlin, Germany; ^2^ European Molecular Biology Laboratory, Hamburg Unit, Hamburg, Germany; ^3^ Centre for Structural Systems Biology, Hamburg, Germany

**Keywords:** *Plasmodium falciparum*, malaria, drug target, transport pathway, transporters and channels, systems biology, calcium homeostasis, nutrient uptake

## Abstract

Malaria remains a leading cause of death and disease in many tropical and subtropical regions of the world. Due to the alarming spread of resistance to almost all available antimalarial drugs, novel therapeutic strategies are urgently needed. As the intracellular human malaria parasite *Plasmodium falciparum* depends entirely on the host to meet its nutrient requirements and the majority of its transmembrane transporters are essential and lack human orthologs, these have often been suggested as potential targets of novel antimalarial drugs. However, membrane proteins are less amenable to proteomic tools compared to soluble parasite proteins, and have thus not been characterised as well. While it had been proposed that *P. falciparum* had a lower number of transporters (2.5% of its predicted proteome) in comparison to most reference genomes, manual curation of information from various sources led to the identification of 197 known and putative transporter genes, representing almost 4% of all parasite genes, a proportion that is comparable to well-studied metazoan species. This transporter list presented here was compiled by collating data from several databases along with extensive literature searches, and includes parasite-encoded membrane-resident/associated channels, carriers, and pumps that are located within the parasite or exported to the host cell. It provides updated information on the substrates, subcellular localisation, class, predicted essentiality, and the presence or absence of human orthologs of *P. falciparum* transporters to quickly identify essential proteins without human orthologs for further functional characterisation and potential exploitation as novel drug targets.

## Introduction

To sustain rapid growth within human red blood cells, *Plasmodium falciparum* requires sufficient nutrients and electrolytes for its active metabolism. Therefore, the parasite expresses a wide range of transport proteins to acquire substrates and efflux metabolites. As the majority of these carriers, channels, and pumps are predicted to be essential during intraerythrocytic stages (Martin, 2020) and have no identified human orthologs, these could be exploited as targets of novel drugs (Ludin et al., 2012). Due to the emergence of parasite resistance to most available antimalarials, new therapeutic strategies are urgently needed (Plowe, 2022). There are many reports on transporters associated with drug resistance (Cowell and Winzeler, 2019; Martin, 2020; Murithi et al., 2021; Shafik et al., 2022), and advances in the development of drugs that target solute transporters were recently reviewed (Belete, 2020; Monteiro Júnior et al., 2022). Here, an extended list of *P. falciparum* transport proteins is presented with many new additions and updated information on transporter localisation and essentiality based on experimental evidence and orthology inference.

The last two transporter lists were published in 2020 and 2016 and contained 117 (Martin, 2020) and 139 (Weiner and Kooij, 2016) proteins, corresponding to 2.2% and 2.6% of the predicted *P. falciparum* proteome, respectively. The localisation within the parasite-infected host cell was not indicated for all of these, as microscopic examination after endogenous tagging with fluorescent proteins or staining using specific antibodies was not conducted for all transporters. However, precise knowledge of the location of a transport protein and its orientation in the membrane is paramount for understanding its function and the dynamics of solute transport processes between cellular compartments. Therefore, the list presented here contains new information on subcellular localisation and function based on results from recent microscopy experiments (Edaye and Georges, 2015; Haase et al., 2021; Murithi et al., 2021; Wichers et al., 2021; Ahiya et al., 2022; Wichers et al., 2022), solubility assays, immunoprecipitation, proximity-dependent biotinylation or subcellular fractionation followed by immunoblot or proteomic analyses (Boucher et al., 2018; Balestra et al., 2021; Bullen et al., 2022), functional and structural studies (Shafik et al., 2020; Beck and Ho, 2021), the presence of targeting signals (Sayers et al., 2018; van Esveld et al., 2021), and Gene Ontology (GO) annotations (Blake et al., 2015). In addition, data on essentiality of *P. falciparum* genes are usually based on a large piggyBac screen (Zhang et al., 2018) that is known to contain some false-positive and false-negative results (Martin, 2020), highlighting the need for verification by other studies. Thus, results from the latest publications (Jiang et al., 2020; Swift et al., 2020; Oberstaller et al., 2021; Wichers et al., 2022) were included in the list along with information on the presence or absence of human orthologs, as this is important for therapeutic development and was not systematically specified previously. Of note, this mini review focuses mainly on asexual blood-stage parasites and also contains recent data on other stages, as transporters are likely important throughout the life cycle.

*Plasmodium* gene annotations are still incomplete with a large proportion of genes completely lacking characterisation of their function and localisation or only having sparse functional annotation deduced by orthology (Böhme et al., 2019). The lower number of genes representing the malaria transportome reported in earlier studies may be due to the lack of conventional transmembrane domains in some *P. falciparum* transporters (Desai, 2012) and difficult analysis by mass spectrometry. The reduced number of detected peptides (Lu et al., 2021) stems both from the typically low protein amounts extracted from parasite culture that are subjected to subcellular fractionation or immunoprecipitation and from the fact that membrane proteins such as transporters are less amenable to proteomics compared to soluble proteins. This has resulted in the conclusion that *P. falciparum* may have a reduced set of transporters compared to metazoan reference genomes (Weiner and Kooij, 2016; Martin, 2020).

Here, additional putative transporters were detected by compiling data from several databases (Aurrecoechea et al., 2009; Blake et al., 2015; Saier et al., 2016; Elbourne et al., 2017) and the literature. This mini review also covers newly identified putative calcium transporters (Balestra et al., 2021; Gupta et al., 2022), as calcium homeostasis is thought to be critical for all parasite stages (Brochet and Billker, 2016) and likely a promising drug target (Gupta et al., 2022). However, the molecular identity of most of the transporters involved in calcium transport has remained unclear (Lourido and Moreno, 2015), with contrasting results and conclusions regarding their substrates and subcellular localisation as well as the cellular compartment used for calcium storage (Brochet and Billker, 2016). The manually curated list of 197 transporter genes presented here represents almost 4% of 5720 P*. falciparum* 3D7 genes, of which 5318 are protein-coding (Aurrecoechea et al., 2009), a proportion that is comparable to the 3 – 5% reported for well-studied metazoan species (Elbourne et al., 2017). It includes the most recent published data and provides an updated overview on the substrates, localisation, function, classification, essentiality, and human orthologs of *P. falciparum* transporters and may serve as a basis for improved annotations of transporter genes and further functional characterisation of potential drug targets.

## Approaches for Transport Protein Identification and Compilation of a Comprehensive List

Whole-genome sequencing, genome-wide searches and comparative genomics enabled the detection and fast annotation of many *P. falciparum* transporter genes by assigning functions that are computationally inferred from orthology across hundreds of species, facilitating functional characterisation at a large scale. However, molecular pathways and mechanisms that occur in parasites can differ tremendously from model organisms (Woo et al., 2015), and some known *Plasmodium* transporters are genus-specific and/or lack conventional transmembrane domains (Desai, 2012). Thus, function predictions based on the presence of protein features and on orthology inference harbour the possibility of incomplete or incorrect annotations. For example, PF3D7_1368200 was annotated as “ABC transporter E family member 1, putative (ABCE1)” due to its ATP-binding cassette that similar to that of ABC transporters (Koenderink et al., 2010). However, it is unlikely to be a transporter because of its function in RNA processing (Mather et al., 2007; Sinha et al., 2021), demonstrating the need for manual curation of GO terms and gene annotations.

The existing transporter list published in 2020 (Martin, 2020) was extended by collating data from various sources. Therefore, a table of 123 transport proteins from the *P. falciparum* strain 3D7 (genome version 3.0) with information on substrates, transporter classes and families was downloaded from http://www.membranetransport.org/transportDB2/index.html (Elbourne et al., 2017). Additional transporters associated with the GO term “transmembrane transporter activity” (GO:0022857) (Blake et al., 2015), mentioned on Malaria Parasite Metabolic Pathways (https://mpmp.huji.ac.il/maps/transporters.html) (Ginsburg and Tilley, 2011) or in research articles were included. For example, *Pf*TMCO1 (transmembrane and coiled-coil domain-containing protein, PF3D7_1362300), identified based on orthology to proteins in other protozoan parasites (Gupta et al., 2022), was added. In contrast, glideosome-associated protein 40 (*Pf*GAP40, PF3D7_0515700) and rhoptry protein *Pf*ROP14 (PF3D7_0613300) were removed, as new data on their function and localisation suggest that these are not transporters (Anantharaman et al., 2007; Zuccala et al., 2012; Ferreira et al., 2020).

As different names were sometimes used for the same protein (Weiner and Kooij, 2016; Staines et al., 2017; Martin, 2020), all alternative names found in the literature are mentioned in the table for clarification (**Table 1**). Transporter localisation, substrates and functions are indicated as in Martin, (2020) and predicted gene essentiality according to Zhang et al. (2018), unless stated otherwise. Transporter classes were assigned according to the Transport Classification Database (TCDB) (Saier et al., 2016) and if the transporter family was unknown, it was assigned according to the top TCDB blast hit (http://www.tcdb.org/progs/blast.php) based on sequence similarity to known transport proteins (Altschul et al., 1997). Data on the presence of human orthologs was retrieved from https://mpmp.huji.ac.il/maps/orth_hsap.html (Ginsburg and Tilley, 2011), a list compiled using recent publications. The existence of human orthologs was further verified using the TCDB protein blast.

**Table 1:** Characteristics of known and putative *P. falciparum* transport proteins.

Gene ID	Product	Substrate and function	Family	Localisation		Essential	Human ortholog
PF3D7_1227200	K1, Kch1	voltage-gated potassium channel	1.A.1	e - EPM (Waller et al., 2008)		b - yes	yes
PF3D7_1465500	K2, Kch2	voltage-gated potassium channel	1.A.1	e - PPM (Waller et al., 2008)		b - no	yes
PF3D7_1436100	NIC	putative K^+^ channel (Ginsburg and Tilley, 2011)	1.A.1	c - PPM		b - yes	no
PF3D7_1132800	AQP	channel for water, glycerol and polyols	1.A.8	e - PPM (Swearingen et al., 2016)		b - yes	yes
PF3D7_1438100	SEC62	protein import in complex with Sec61 (Marapana et al., 2018)	1.A.15	e - ER (Marapana et al., 2018)		b - yes	yes
PF3D7_1250200	CSC, CSC1	calcium-activated stress-gated channel for Ca^2+,^ K^+^ and Na^+^	1.A.17	c - PPM (Blake et al., 2015)		b - yes	yes
PF3D7_1107900	MSCS	putative mechanosensitive anion channel	1.A.23	c - PPM? (Blake et al., 2015)		b - no	no
PF3D7_1120300	MIT1	magnesium/nickel/cobalt ion channel (Ginsburg and Tilley, 2011)	1.A.35	c - mitochondrion (van Esveld et al., 2021)		b - no	yes
PF3D7_1304200	MIT2	magnesium/nickel/cobalt ion channel (Ginsburg and Tilley, 2011)	1.A.35	c - mitochondrion (Blake et al., 2015)		b - yes	no
PF3D7_1427600	MIT3	magnesium/nickel/cobalt ion channel (Ginsburg and Tilley, 2011)	1.A.35	c - mitochondrion (Blake et al., 2015)		b - no	yes
PF3D7_1333800	ICln	anion channel	1.A.47	c - PPM		b - no	no
PF3D7_1439000	CTR1	copper channel	1.A.56	e - EPM, PPM		b - yes	no
PF3D7_1421900	CTR2	copper channel	1.A.56	c - apicoplast		b - yes	no
PF3D7_0306700	MMgT, EMC5	magnesium channel	1.A.67	c - ER		b - yes	no
PF3D7_0302500	CLAG3.1, RhopH1	PSAC/RhopH complex components for nutrient uptake (anions/organic cations)	1.A.91.1.1	e - EPM		b - no	no
PF3D7_0302200	CLAG3.2, RhopH1			e - EPM		b - no	no
PF3D7_0220800	CLAG2			c - EPM		b - no	no
PF3D7_0831600	CLAG8			c - EPM		b - no	no
PF3D7_0935800	CLAG9			c - EPM		b - no (Nacer et al., 2011)	no
PF3D7_0929400	RhopH2			e - EPM		b - yes	no
PF3D7_0905400	RhopH3			e - EPM		b - yes	no
PF3D7_1362300	TMCO1	Ca^2+^ channel, prevents ER overfilling? (Wang et al., 2016)	1.A.106	c - ER? (Blake et al., 2015)		unknown	yes
PF3D7_1432100	OMPP, VDAC	solute channel	1.B.8.5.2	c - mitochondrion (Blake et al., 2015)		unknown	no
PF3D7_0823700	TOM7	components of TOM complex for protein import across outer membrane (Sheiner and Soldati-Favre, 2008; Schmidt et al., 2010)	1.B.8	c - mitochondrion (Schmidt et al., 2010)		b - yes	no
PF3D7_0524700	TOM22			e - mitochondrion (van Dooren et al., 2006)		b - yes	no
PF3D7_0617000	TOM40			e - mitochondrion (Das et al., 2017)		b - yes	no
PF3D7_0408700	PLP1, PPLP1	erythrocyte permeabilisation and rupture (Garg et al., 2013)	1.C.39	e - EPM (Garg et al., 2013)		b - no, s - yes (Yang et al., 2017)	no
PF3D7_1216700	PLP2, PPLP2	erythrocyte permeabilisation and rupture (Wirth et al., 2014)	1.C.39	e - EPM (Wirth et al., 2014)		b - no, g - yes (Wirth et al., 2014)	no
PF3D7_0923300	PLP3, PPLP3	host cell permeabilisation and rupture (Sassmannshausen et al., 2020)	1.C.39	c - host cell membrane (Sassmannshausen et al., 2020)		unknown	no
PF3D7_0819400	PLP4, PPLP4	rupture of mosquito midgut epithelial cells (Wirth et al., 2015)	1.C.39	e - host cell membrane (Sassmannshausen et al., 2020)		b - no, o - yes (Wirth et al., 2015)	no
PF3D7_0819200	PLP5, PPLP5	host cell permeabilisation and rupture (Sassmannshausen et al., 2020)	1.C.39	c - host cell membrane (Sassmannshausen et al., 2020)		b - yes	no
PF3D7_1331500		putative calcium channel (Gupta et al., 2022)	1.C.105	c - PPM? (Blake et al., 2015)		unknown	yes
PF3D7_1234600	TOC75	protein import across 2^nd^ inner membrane (Agrawal and Striepen, 2010)	1.C.105	c - apicoplast (Boucher et al., 2018)		b - yes	no
PF3D7_0104100	E140, MPMP	unknown	1.C.105	c - PPM? (Blake et al., 2015)		b - yes	no
PF3D7_1455400	HlyIII	forms pore (~3.2 nm) for solutes and ions	1.C.113	e - EPM		b - yes	no
PF3D7_0204700	HT1	imports glucose and fructose	2.A.1.1	e - PPM		b - yes	yes
PF3D7_0516500	MFS1, MDT	putative metabolite/drug transporter	2.A.1.2	unknown		b - no	yes
PF3D7_0916000	MFS2	putative sugar transporter	2.A.1.1	unknown		b - no	yes
PF3D7_0919500	MFS3	putative sugar transporter	2.A.1.1	e - PPM? (Swearingen et al., 2016), c - mitochondrion (Blake et al., 2015)		b - no	yes
PF3D7_1203400	MFS4	putative transporter	2.A.1	unknown		b - no	no
PF3D7_1428200	MFS5	putative metabolite transporter	2.A.1	unknown		b - no	no
PF3D7_1440800	MFS6	H^+^ import, metabolite/drug export	2.A.1	e - apicoplast		b - no	no
PF3D7_1117000	P115	unknown	2.A.1	c - PPM (Blake et al., 2015)		b - no	no
PF3D7_0614300	MFR1	putative organic anion transporter	2.A.1.2	unknown		b - no	no
PF3D7_0104700	MFR2, ApiAT9	putative amino acid transporter	2.A.1	e - PPM (Wichers et al., 2021)		b - no	no
PF3D7_0312500	MFR3, ApiAT10	putative amino acid transporter	2.A.1	e - PPM (Wichers et al., 2021)		b - no	no
PF3D7_0914700	MFR4, ApiAT2	putative amino acid transporter	2.A.1	e - PPM (Wichers et al., 2021)		b - no	no
PF3D7_1129900	MFR5, ApiAT4	putative amino acid transporter	2.A.1	e - PPM (Wichers et al., 2021)		b - no	no
PF3D7_0104800	NPT1, ApiAT8	putative amino acid transporter	2.A.1	e - PPM (Wichers et al., 2021)		b - no	no
PF3D7_0210300	MCT1, MCP1	exports monocarboxylate	2.A.1	c - PPM		b - yes	yes
PF3D7_0926400	MCT2, MCP2	exports organic solutes, imports H^+^	2.A.1	e - apicoplast (Boucher et al., 2018)		b - no	no
PF3D7_1036800	ACT, AT, AT1	imports acetyl-CoA, exports CoA	2.A.1.25	e - ER		b - no	yes
PF3D7_1104800	UMF	pantothenate:H^+^ import	2.A.1.63	c - PPM		b - yes	no
PF3D7_0206200	TFP1, PAT	pantothenate:H^+^ import (Ginsburg and Tilley, 2011)	2.A.1.66	e - PPM		b - no	yes
PF3D7_0529200	GPH	putative sugar:cation symporter	2.A.2	unknown		b - no	no
PF3D7_0715900	CDF, ZIP3	Zn^2+^ import? (Huang et al., 2014)	2.A.4	e - cytoplasmic vesicle (Wichers et al., 2022)		b - no	yes
PF3D7_0609100	ZIP1	Zn^2+^ import? (Ginsburg and Tilley, 2011)	2.A.5	e - PPM (Wichers et al., 2022)		b - no	yes
PF3D7_1022300	ZIPCO, ZIP2	Zn^2+^/Fe^2+^ import into cytosol	2.A.5	c - PPM? (Blake et al., 2015)		b - no	yes
PF3D7_0107500	NCR1, NPC1R	cholesterol/sterol/lipid export, H^+^ import	2.A.6.6	e - PPM		b - yes	yes
PF3D7_0715800	DMT1	organic solute transport	2.A.7.3	c - apicoplast		b - no	yes
PF3D7_0716900	DMT2	IPP export	2.A.7	e - apicoplast		b - yes	no
PF3D7_0709000	CRT	drug/peptide:H^+^ export	2.A.7.3	e - DV		b - yes	no
PF3D7_0508300	TPT, _o_TPT, _o_pPT	PEP/3GP import, P_i_ export	2.A.7.9	e - apicoplast		b - yes	yes
PF3D7_0530200	PPT, _i_TPT, _i_pPT	PEP/3GP import, P_i_ export	2.A.7.9	e - apicoplast		b - yes (Swift et al., 2020)	yes
PF3D7_1218400	TPT3	putative organic phosphate ester:P_i_ antiporter	2.A.7.9	unknown		b - no	yes
PF3D7_0505300	NGT	UDP-N-acetylglucosamine import, UMP export	2.A.7.10	c - Golgi		b - no	yes
PF3D7_1113300	UGT	UDP-galactose/UDP-glucose import, UMP export	2.A.7.11	e - ER		b - yes	yes
PF3D7_0212000	GFT	GDP-fucose import, GMP export	2.A.7.16	c - Golgi		b - yes	yes
PF3D7_0522600	NIPA	Mg^2+^ import	2.A.7.25	e - PPM		b - yes	yes
PF3D7_0629500	AAT1	transports Ile, Leu, Met	2.A.18	c - PPM, DV		b - yes	yes
PF3D7_1208400	AAT2	transports amino acids, GABA	2.A.18	c - PPM		b - no	yes
PF3D7_1231400	AAAP3, ICM1	transports Ile, Leu, Met or Ca^2+^ (Balestra et al., 2021)	2.A.18	unknown		b - yes	no
PF3D7_0603500	CAX, CHA	imports H^+^, exports Ca^2+^/Mg^2+^/Mn^2+^	2.A.19	e - mitochondrion (Rotmann et al., 2010)		b - no	no
PF3D7_1340900	PiT	imports phosphate and Na^2+^ into cytosol	2.A.20	e - PPM		b - yes	yes
PF3D7_0209600	NSS1	putative amino acid transporter	2.A.22	c - PPM (Blake et al., 2015)		b - yes	yes
PF3D7_0515500	GEP1, NSS2	neurotransmitter:Na^2+^ symport (Ginsburg and Tilley, 2011)	2.A.22	c - cytoplasmic vesicle (Jiang et al., 2020)		b - no	no
PF3D7_1132500	NSS3	amino acid/GABA transport	2.A.22	c - PPM		b - no	yes
PF3D7_0714100	MAATS1	export of H^+^ and amino acids (Ginsburg and Tilley, 2011)	2.A.22	unknown		b - no	yes
PF3D7_1368700	TPC, DNC	thiamine pyrophosphate import, nucleotide export	2.A.29	c - mitochondrion		b - yes	yes
PF3D7_0905200	MRS3, MC5	putative Fe^2+^ importer (Blake et al., 2015)	2.A.29	c - mitochondrion		b - yes	yes
PF3D7_0407500	MTM1, MC3	unknown	2.A.29	c - mitochondrion		b - yes	yes
PF3D7_1241600	SAMC, PET8	imports S-adenosylmethionine, exports S-adenosylhomocysteine	2.A.29	e - mitochondrion		b - yes	yes
PF3D7_0108400	MME1, MC1	unknown	2.A.29	c - mitochondrion		b - no	yes
PF3D7_0108800	AMC1, MC2	unknown	2.A.29	c - mitochondrion		b - yes	no
PF3D7_0811100	AMC2, MC4	unknown	2.A.29	c - mitochondrion		b - no	yes
PF3D7_0908800	AMC3, MC6	unknown	2.A.29	c - mitochondrion		b - yes	yes
PF3D7_1037300	AAC1, ADT	ADP/ATP antiporter (Blake et al., 2015)	2.A.29	e - mitochondrion (Hatin et al., 1992)		b - yes	yes
PF3D7_1004800	AAC2, PAAC	ADP/ATP antiporter (Blake et al., 2015)	2.A.29	c - mitochondrion (van Esveld et al., 2021)		b - yes	yes
PF3D7_1223800	COC, YHM2	imports oxoglutarate, exports citrate	2.A.29	c - mitochondrion		b - no	yes
PF3D7_0823900	DTC, OMT	imports dicarboxylate, exports tricarboxylate	2.A.29	e - mitochondrion		b - yes	yes
PF3D7_1202200	MPC, PIC, PIC2	P_i_:H^+^ import	2.A.29	c - mitochondrion		b - no	yes
PF3D7_1303500	NHE	H^+^ import into cytosol in exchange for Na^+^	2.A.36	c - PPM (Blake et al., 2015)		b - no	yes
PF3D7_0924500		putative Na^+^:H^+^ exchanger (Saier et al., 2016)	2.A.36	unknown		b - yes	yes
PF3D7_0827700	MgT1	Mg^2+^:H^+^ antiporter (Blake et al., 2015)	2.A.36	unknown		b - no	yes
PF3D7_1135000		unknown	2.A.43	c - apicoplast (Boucher et al., 2018)		unknown	no
PF3D7_0316600	FNT	lactate/formate and H^+^ release from cytosol	2.A.44	e - PPM, DV		b - no	no
PF3D7_1471200	SuIP	inorganic anion antiporter	2.A.53	e - PPM		b - yes	yes
PF3D7_0523800	NRAMP2, NRAMP, FVRT1	Fe^2+^/ Mn^2+^:H^+^ export	2.A.55	e - DV (Wichers et al., 2022)		b - yes	yes
PF3D7_1347200	NT1, ENT1	purine base import	2.A.57	e - PPM		b - yes	no
PF3D7_0824400	NT2, ENT2	nucleoside/nucleobase import	2.A.57	e - ER		b - no	no
PF3D7_1469400	NT3, ENT3	putative nucleoside transporter	2.A.57	unknown		b - no	no
PF3D7_0103200	NT4, ENT4	adenine/adenosine import	2.A.57	c - PPM		b - yes	no
PF3D7_0212800	MATE	putative organic solute:Na^+^/H^+^ antiporter	2.A.66.1	unknown		b - no	yes
PF3D7_0828600	FT1	imports pABA and folates	2.A.71	e - PPM		b - no	no
PF3D7_1116500	FT2	imports pABA, folates, 5-methyltetrahydrofolate	2.A.71	e - PPM		b - no	no
PF3D7_1223700	VIT	imports Fe^2+^ for detoxification, exports H^+^	2.A.89	unknown		b - no	no
PF3D7_0417300	LETM1	imports H^+^, exports Ca^2+^/K^+^	2.A.97	c - mitochondrion (van Esveld et al., 2021)		b - yes	yes
PF3D7_1340800	MPC1	pyruvate:H^+^ importer	2.A.105	c - mitochondrion		b - yes	yes
PF3D7_1470400	MPC2	pyruvate:H^+^ importer	2.A.105	c - mitochondrion		unknown	yes
PF3D7_1033000	HPR1, AMC4	unknown	2.A.123	c - mitochondrion? (van Esveld et al., 2021)		b - yes	no
PF3D7_0216600	SWEET	putative glucose/galactose transporter	2.A.123	c - ER/Golgi		b - yes	yes
PF3D7_0305300		unknown	2.A.123	unknown		b - no	no
PF3D7_0523000	MDR1, ABCB1, Pgh1	active drug and solute import (Friedrich et al., 2014)	3.A.1.201	e - DV (Papalexis et al., 2001)		b - yes	yes
PF3D7_1447900	MDR2, ABCB2	active Cd^2+^ extrusion from cytosol	3.A.1.210	e - PPM, DV		b - no (van der Velden et al., 2015)	yes
PF3D7_1145500	MDR3, ABCB3	active peptide efflux	3.A.1.209	e - apicoplast (Boucher et al., 2018)		b - no	yes
PF3D7_0302600	MDR4, ABCB4	active peptide/heavy metal cation transport	3.A.1.209	e - apicoplast		b - no	yes
PF3D7_1339900	MDR5, ABCB5	active solute export	3.A.1.201	e - PPM		b - no	yes
PF3D7_1352100	MDR6, ABCB6, Atm1	active glutathione trisulfide efflux	3.A.1.210	c - mitochondrion, apicoplast		b - yes	yes
PF3D7_1209900	MDR7, ABCB7	active peptide efflux	3.A.1.209	c - mitochondrion		b - no	yes
PF3D7_0112200	MRP1, ABCC1	active export of drugs and glutathione conjugates	3.A.1.208	e - PPM		b - no	yes
PF3D7_1229100	MRP2, ABCC2	active export of glutathione conjugates	3.A.1.208	e - PPM		b - no	yes
PF3D7_0813700	ABCF1	heme import? (Blake et al., 2015)	3.A.1	e - apicoplast (Boucher et al., 2018)		b - yes	yes
PF3D7_1426500	ABCG, ABCG1, ABCG2	putative cell metabolite exporter (Edaye and Georges, 2015)	3.A.1.204	e - PPM (Edaye and Georges, 2015)		b - no	yes
PF3D7_0319700	ABCI3	active solute transport (Murithi et al., 2021)	3.A.1	e - cytoplasmic vesicle (Murithi et al., 2021)		unknown	yes
PF3D7_0810200	ABCK1	active peptide efflux (Ginsburg and Tilley, 2011)	3.A.1	c - mitochondrion (van Esveld et al., 2021)		b - yes	yes
PF3D7_1004600		drug transport? (Park et al., 2012)	3.A.1	unknown		b - no	no
PF3D7_0812900		drug transport? (Park et al., 2012)	3.A.1	unknown		b - no	no
PF3D7_1434000	CAF16	putative ABC transporter (Blake et al., 2015)	3.A.1	unknown		b - yes	yes
PF3D7_0614900		unknown	3.A.1	c - PPM (Blake et al., 2015)		b - no	yes
PF3D7_1144700	TIC20	protein import across innermost membrane (Agrawal and Striepen, 2010)	3.A.1	c - apicoplast (Boucher et al., 2018)		b - yes	no
PF3D7_1121600	EXP1	pore for solutes < 1.4 kDa with EXP2 (Mesén-Ramírez et al., 2019)	3.A.1	e - PVM (Mesén-Ramírez et al., 2019)		b - yes (Maier et al., 2008)	no
PF3D7_0217100	ATPα, F_1_ α	H^+^-importing ATP synthase subunits	3.A.2	e - mitochondrion		b - yes	yes
PF3D7_1235700	ATPβ, F_1_ β					b - no	yes
PF3D7_1311300	ATPγ, F_1_ γ					b - yes	yes
PF3D7_1147700	ATPδ, F_1_ δ					b - no	no
PF3D7_0715500	ATPϵ, F_1_ ϵ					b - no	no
PF3D7_1310000	OSCP					b - yes	yes
PF3D7_0719100	F_o_ a					b - yes	no
PF3D7_1125100	F_o_ b					b - yes	no
PF3D7_0705900	F_o_ c					b - yes	yes
PF3D7_0311800	F_o_ d					b - yes	no
PF3D7_1311900	vapA, V_1_ subunit A	V-ATPase subunits: active H^+^ export from cytosol	3.A.2	e - PPM, DV, cytoplasmic vesicle (Hayashi et al., 2000)		b - yes	yes
PF3D7_0406100	vapB, V_1_ subunit B					b - yes	yes
PF3D7_0106100	vapC, V_1_ subunit C					b - yes	yes
PF3D7_1341900	vapD, V_1_ subunit D					b - yes	yes
PF3D7_0934500	vapE, V_1_ subunit E					b - yes	yes
PF3D7_1140100	vapF, V_1_ subunit F					b - no	yes
PF3D7_1323200	vapG, V_1_ subunit G					b - yes	no
PF3D7_1306600	vapH, V_1_ subunit H					b - yes	yes
PF3D7_0806800	V_o_ subunit a					b - yes	yes
PF3D7_0519200	V_o_ subunit c, 16-kDa proteolipid					b - no	yes
PF3D7_1354400	V_o_ subunit c", 21-kDa proteolipid					b - yes	yes
PF3D7_1464700	V_o_ subunit d, C/AC39					b - yes	yes
PF3D7_0721900	V_o_ subunit e					b - yes	no
PF3D7_0516100	ATP1	extrusion of inorganic cations from cytosol	3.A.3	e - PPM, DV		b - no	yes
PF3D7_1219600	ATP2	putative phospholipid flippase	3.A.3	c - PPM		b - yes	yes
PF3D7_0504000	ATP3	active Mg^2+^ transport	3.A.3	c - apicoplast		b - yes	yes
PF3D7_1211900	ATP4	H^+^ import, Na^+^ export	3.A.3	e - PPM		b - yes	yes
PF3D7_0106300	ATP6	active Ca^2+^ import for storage	3.A.3	c - ER		b - yes	yes
PF3D7_0319000	ATP7	putative phospholipid flippase	3.A.3	c - PPM (Blake et al., 2015)		b - no	yes
PF3D7_1223400	ATP8	putative phospholipid flippase	3.A.3	c - PPM		b - yes	yes
PF3D7_1348800	ATP9	active Ca^2+^ import?	3.A.3	c - DV?		b - no	yes
PF3D7_0727800	ATP10	active Mn^2+^ transport	3.A.3	c - apicoplast		b - yes	yes
PF3D7_1468600	ATP11	putative phospholipid flippase	3.A.3	c - PPM (Blake et al., 2015)		b - no	yes
PF3D7_0904900	CuTP	active Cu^2+^ export	3.A.3	e - EPM, PPM		b - no	yes
PF3D7_1138400	GCα	phospholipid flippase	3.A.3	c - cytoplasmic vesicle (Jiang et al., 2020)		b - yes (Taylor et al., 2008)	yes
PF3D7_1360500	GCβ	phospholipid flippase	3.A.3	c - PPM		b - no	yes
PF3D7_1346100	SEC61α	components of ER translocon for import of proteins destined for export, interact with SEC62 (Marapana et al., 2018)	3.A.5	e - ER (Marapana et al., 2018)		b - no	yes
PF3D7_0821800	SEC61β					b - no	yes
PF3D7_0210000	SEC61γ					b - yes	yes
PF3D7_1318800	SEC63					b - yes	yes
PF3D7_0724400	TIM14, PAM18	components of TIM23/PAM complex for protein import across inner membrane (Sheiner and Soldati-Favre, 2008; Schmidt et al., 2010)	3.A.8	c - mitochondrion (van Esveld et al., 2021)		b - yes	yes
PF3D7_0513500	TIM16, PAM16					unknown	no
PF3D7_1434700	TIM17					b - yes	yes
PF3D7_1356200	TIM23					b - yes	no
PF3D7_1125400	TIM44					b - yes	yes
PF3D7_0726900	TIM50					b - yes	yes
PF3D7_0627400	TIM22	protein import across inner membrane (Sheiner and Soldati-Favre, 2008; Schmidt et al., 2010)	3.A.8	c - mitochondrion (van Esveld et al., 2021)		b - yes	yes
PF3D7_1456800	VP1	active H^+^ export	3.A.10	e - PPM (Ahiya et al., 2022)		b - yes	no
PF3D7_1235200	VP2	putative Ca^2+^-dependent H^+^ export from cytosol	3.A.10	e - PPM, cytoplasmic vesicles (Marchesini et al., 2000)		b - no	no
PF3D7_0810400	AQP2	water channel (Blake et al., 2015)	3.A.16	c - PPM (Blake et al., 2015)		b - no	no
PF3D7_0314300	Der1-1	protein import across periplastid membrane (Spork et al., 2009)	3.A.25.2.1	e - apicoplast (Spork et al., 2009)		b - yes	no
PF3D7_1452300	Der1-2	protein import across periplastid membrane (Spork et al., 2009)	3.A.25.2.1	e - apicoplast (Spork et al., 2009)		unknown	yes
PF3D7_0216800		unknown	3.A.25	unknown		b - yes	yes
PF3D7_0315700		unknown	3.A.25	unknown		b - no	no
PF3D7_1471100	EXP2	PTEX core components for protein export (Beck and Ho, 2021), EXP2 also functions as a pore for solutes < 1.4 kDa together with EXP1 (Garten et al., 2018; Mesén-Ramírez et al., 2019)	3.A.26.1.1		e - PVM (de Koning-Ward et al., 2009)	b - yes	no
PF3D7_1436300	PTEX150					b - yes (de Koning-Ward et al., 2009)	no
PF3D7_1116800	HSP101					b - yes	yes
PF3D7_1404600	ACα	putative K^+^ channel	8.A.85	unknown		b - no	no
PF3D7_1022700	PLSCR	phospholipid scramblase (Haase et al., 2021)	9.A.36	e - parasite periphery (Haase et al., 2021)		b - no	no
PF3D7_1332100		putative transporter	9.B.14	unknown		b - no	no
PF3D7_0530500		putative transporter	9.B.14	unknown		b - no	no
PF3D7_0628400		unknown	9.B.14	unknown		b - no	no
PF3D7_1135300	PMRT1	unknown	9.B.14	e - PPM (Wichers et al., 2022)		b, g - yes (Wichers et al., 2022)	no
PF3D7_1022200	FBT	putative metabolite/vitamin transporter (Ginsburg and Tilley, 2011)	9.B.14	unknown		b - yes	no
Pf3D7_0321900	CARL	unknown	9.B.314	e - cis-Golgi (LaMonte et al., 2016)		b - no	yes
PF3D7_0824700	LMF1	putative transporter	9.B.365.5.1	c - ER (Blake et al., 2015)		b - no	yes

Substrates, functions, and localisations are indicated as in Martin (2020), unless stated otherwise. Known or putative localisation refers to the site of active function of the transport protein regardless of its trafficking route, as evidenced either by experimental data (e) or computational analysis (c). DV: digestive vacuole, EPM, erythrocyte plasma membrane; PPM, parasite plasma membrane; PVM, parasitophorous vacuole membrane. Transporter families were assigned according to the Transport Classification Database (Saier et al., 2016). 1: channels and pores, 1.A: α-type channels, 1.B: β-barrel porins, 1.C: pore-forming toxins. 2: electrochemical potential-driven transporters, 2.A: porters (uniporters, symporters, antiporters), 3: primary active transporters, 3.A: P-P-bond-hydrolysis-driven transporters, 8: accessory factors involved in transport, 8.A: auxiliary transport proteins, 9: incompletely characterised transport systems, 9.A: recognised transporters of unknown biochemical mechanism, 9.B: putative transport proteins. Predicted gene essentiality refers to Zhang et al. (2018), unless another reference is given. The tested life cycle stages are indicated as b, asexual blood stage; g, gametocytes; o, ookinetes; s, sporozoites. Information on the presence of human orthologs is listed according to https://mpmp.huji.ac.il/maps/orth_hsap.html (Ginsburg and Tilley, 2011).

In total, 197 transport proteins were identified (**Table 1**), with some of these forming a complex, e.g. the *Plasmodium* Translocon of EXported proteins (PTEX), consisting of three core components (de Koning-Ward et al., 2009; Beck and Ho, 2021). Protein complex components residing in or associated with the respective membrane that are required for substrate translocation were included, whereas accessory and auxiliary subunits were excluded. For clarity, only the likely site of active transport is indicated for each protein, although it might be detectable in other subcellular compartments during trafficking.

## Calcium Transport Proteins as Potential Drug Targets

**Figure 1:**
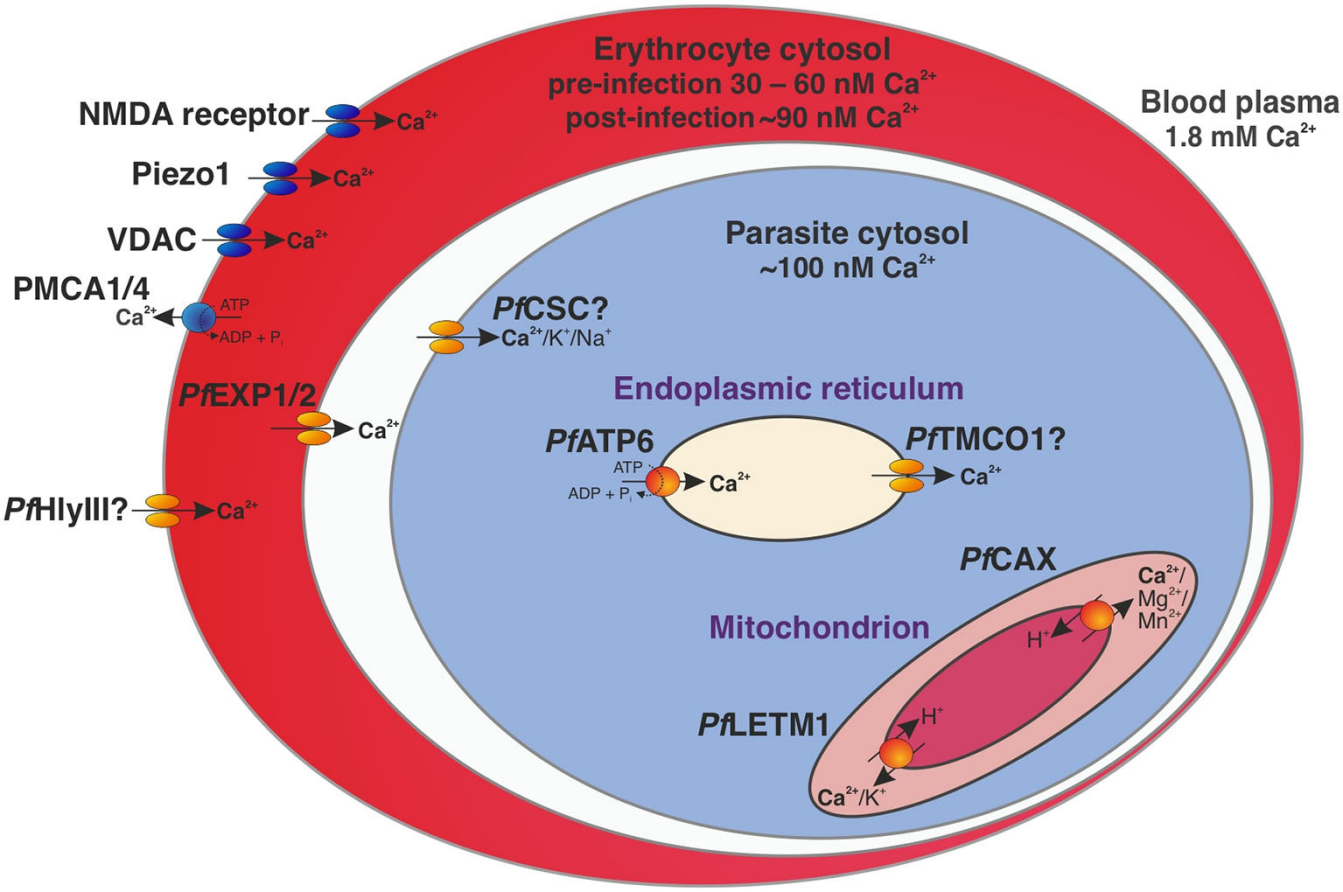
Calcium homeostasis in a trophozoite-stage *P. falciparum-*infected erythrocyte. Under resting conditions, the concentration of free Ca^2+^ is ~1.8 mM in the blood plasma, 30 – 60 nM in cytosol of an uninfected erythrocyte (Brochet and Billker, 2016), ~90 nM in the cytosol of the infected erythrocyte (Rohrbach et al., 2005), and ~100 nM in the cytosol of *P. falciparum* (Garcia et al., 1996). Transport proteins affecting intracellular calcium concentrations in the parasite-infected erythrocyte include the human P-type plasma membrane Ca^2+^-ATPases (PMCA) 1 and 4, human Piezo1, the erythroid N-methyl D-aspartate (NMDA) receptor, the voltage-dependent anion channel (VDAC) (Kaestner et al., 2020), and likely the parasite-encoded hemolysin III (*Pf*HlyIII) (Moonah et al., 2014). A nutrient pore formed by *Pf*EXP1 and *Pf*EXP2 mediates passage through the parasitophorous vacuole membrane (Garten et al., 2018; Mesén-Ramírez et al., 2019) and the calcium-permeable stress-gated cation channel *Pf*CSC may be responsible for Ca^2+^ entry into the parasite cytosol (Martin, 2020). The SERCA-type Ca^2+^-ATPase *Pf*ATP6 actively imports Ca^2+^ into the endoplasmic reticulum as an intracellular reservoir (Lourido and Moreno, 2015; Martin, 2020), while the putative calcium load-activated calcium channel *Pf*TMCO1 (Gupta et al., 2022) may release ions back into the cytosol to avoid overload (Lourido and Moreno, 2015; Wang et al., 2016). Ca^2+^ efflux from the mitochondrion is likely mediated by the cation/H^+^ antiporters *Pf*CAX (Rotmann et al., 2010) and *Pf*LETM1 (Martin, 2020) *via* secondary active transport. Human-encoded transporters and channels are shown in blue and parasite-encoded proteins in orange.

Calcium homeostasis was chosen as an example for illustrating transport pathways in the *P. falciparum*-infected erythrocyte (**Figure 1**), as Ca^2+^ signalling is known to be critical throughout the parasite life cycle (Brochet and Billker, 2016) and a link between Ca^2+^ uptake and virulence has been proposed in the related parasite *Toxoplasma gondii* (Pace et al., 2014). In fact, Ca^2+^ transporters such as *Pf*ATP6 (PF3D7_0106300) are currently under investigation as novel antimalarial drug targets (Gupta et al., 2022; Monteiro Júnior et al., 2022). While the concentration of free Ca^2+^ is ~1.8 mM in the blood plasma, mature erythrocytes only contain 30 – 60 nM Ca^2+^ (Brochet and Billker, 2016) due to active ion extrusion by the P-type plasma membrane Ca^2+^ ATPases (PMCA) 1 and 4 and slow Ca^2+^ uptake *via* several channels such as Piezo1, the erythroid N-methyl D-aspartate (NMDA) receptor, and the voltage-dependent anion channel (VDAC) (Kaestner et al., 2020).

A malaria parasite that resides within an erythrocyte maintains a cytosolic calcium level of approximately 100 nM by permeabilising its host cell and using a regulatory Ca^2+^ pool (Garcia et al., 1996). Extracellular Ca^2+^ is thought to first pass through a parasite-encoded channel in the erythrocyte plasma membrane (EPM) that is independent of PSAC (plasmodial surface anion channel), thereby increasing the intracellular Ca^2+^ concentration of the infected red blood cell (Zipprer et al., 2014). One candidate for this channel is hemolysin III (*Pf*HlyIII, PF3D7_1455400), which forms an ion-permeable pore of approximately 3.2 nm in EPMs after its release from the parasite digestive vacuole (DV) upon merozoite egress (Moonah et al., 2014). Another potential route of Ca^2+^ entry into the infected erythrocyte is *via* enhanced activity of a host channel induced by the parasite, as suggested for VDAC (Bouyer et al., 2011).

Passage through the parasitophorous vacuole membrane (PVM) likely occurs *via* a nutrient pore for solutes < 1.4 kDa formed by *Pf*EXP1 (PF3D7_1121600) and *Pf*EXP2 (PF3D7_1471100) (Garten et al., 2018; Mesén-Ramírez et al., 2019). The ion may then enter the parasite cytosol *via* a parasite-encoded channel, one candidate being the calcium-permeable stress-gated cation channel *Pf*CSC (PF3D7_1250200) that is activated by high external calcium levels (Martin, 2020). The localisation of this transporter at the PPM was inferred from an ancestral gene (Gaudet et al., 2011) and although this remains to be confirmed experimentally, it seems plausible due to the identification of this protein as an immunoreactive antigen with high serodominance in exposed individuals (Doolan et al., 2008). As *Pf*CSC is highly expressed in sporozoites (Le Roch et al., 2003), its exposure to the immune system may occur at this parasite stage.

Calcium can then be stored in the endoplasmic reticulum upon active import by the SERCA-type Ca^2+^-ATPase *Pf*ATP6 (Lourido and Moreno, 2015; Martin, 2020). In case of Ca^2+^ overload of the ER, the putative calcium load-activated calcium channel *Pf*TMCO1 (Gupta et al., 2022) may become active and release ions into the cytosol (Lourido and Moreno, 2015; Wang et al., 2016). Ca^2+^ efflux from the mitochondrion is likely mediated by the cation/H^+^ antiporters *Pf*LETM1 (PF3D7_0417300) (Martin, 2020) and *Pf*CAX/*Pf*CHA (PF3D7_0603500) in exchange for protons that travel along the H^+^ gradient across the inner mitochondrial membrane (Rotmann et al., 2010).

Another putative intracellular Ca^2+^ pool may consist of acidocalcisomes – small electron-dense vesicles that are conserved from bacteria to humans and contain high concentrations of Ca^2+^, pyrophosphate, polyphosphate, iron, and zinc (Huang et al., 2014). Accordingly, acidocalcisome membranes contain a variety of specific transporters for these substrates across the tree of life (Huang et al., 2014). While many transporters were shown to reside in the acidocalcisome membrane in *Trypanosoma brucei* through proteomic studies and microscopy (Huang et al., 2014), no protein has been definitely localised to these organelles in *P. falciparum* (Magowan et al., 1997; Ruiz et al., 2004). Their low internal pH is likely required for the secondary active import of various ions and thought to be established and maintained by the plant-like H^+^-pump V-ATPase (Wunderlich et al., 2012; de Oliveira et al., 2021). This has yet to be verified experimentally, and there may be differences between parasite species. For example, *Pf*VP1 (PF3D7_1456800), an ortholog of the acidocalcisome marker in *T. brucei* (Huang et al., 2014) and *T. gondii* (Rohloff et al., 2011), was previously suggested to localise to the parasite plasma membrane (PPM), DV and acidocalcisomes in *P. falciparum*, but could only be detected at the PPM by microscopy (Ahiya et al., 2022).

Other proteins that may translocate calcium and whose subcellular localisation has not yet been confirmed are *Pf*ATP9 (PF3D7_1348800), the putative calcium channel PF3D7_1331500, and *Pf*ICM1 (PF3D7_1231400). Elucidating their location and function is an important knowledge gap to be addressed (Kustatscher et al., 2022). Of the aforementioned putative Ca^2+^ transport proteins, *Pf*ICM1 and *Pf*HlyIII may be worth exploring as drug targets due to their predicted essentiality and the absence of human counterparts.

## Conclusions and Future Perspectives

This mini review consolidates data from various databases and provides an up-to-date overview of the subcellular localisation, function, predicted essentiality, and human orthologs of *P. falciparum* transporters for the fast identification of essential parasite transporters without human orthologs that may be promising novel targets for therapeutic development. Many of these candidates localise to the apicoplast, the mitochondrion, or the digestive vacuole, which are known to be “druggable” (Wunderlich et al., 2012; Oberstaller et al., 2021).

Moreover, the new transporter list will improve gene annotations and serve as a basis for further functional characterisation of the proteins. It will also be useful for systems biology approaches as it allows more reliable screening of e.g. genomic, transcriptomic, and proteomic data for *P. falciparum* transporters. The low coverage of the *P. falciparum* membrane proteome that complicates target profiling (Lu et al., 2021) may be overcome by large-scale culturing (Dalton et al., 2012) and more sensitive mass spectrometry techniques (McClure and Williams, 2018). Chemogenomic and transcriptional profiling of mutant-parasite libraries with altered drug sensitivities will further guide the determination of the mechanisms of drug action (Adjalley et al., 2015; Pradhan et al., 2015).

## Author Contributions

The author confirms being the sole contributor of this work and has approved it for publication.

## Funding

JW was supported by the Boehringer Ingelheim Foundation and the European Research Council under the European Union’s Horizon 2020 Research and Innovation Programme (grant agreement 759534).

## Author Disclaimer

The funders had no role in study design, data collection, decision to publish, or preparation of the manuscript.

## Conflict of Interest

The author declares that the research was conducted in the absence of any commercial or financial relationships that could be construed as a potential conflict of interest.

## Publisher’s Note

All claims expressed in this article are solely those of the authors and do not necessarily represent those of their affiliated organizations, or those of the publisher, the editors and the reviewers. Any product that may be evaluated in this article, or claim that may be made by its manufacturer, is not guaranteed or endorsed by the publisher.
